# Inflammatory myofibroblastic tumor of the bladder treated with partial cystectomy: Case report

**DOI:** 10.1016/j.ijscr.2025.111225

**Published:** 2025-03-28

**Authors:** Chale Yohannes Tegegne, Mekuanint Asfaw Yitayew, Chalachew Tenna Alemu, Samuel Fekadu Shiferaw, Admassu Melaku Mikru, Abdiqadir Omer Rabile

**Affiliations:** Addis Ababa University, School of Medicine, Addis Ababa, Ethiopia

**Keywords:** Bladder, Inflammatory myofibroblastic tumor, Transurethral resection of bladder tumor, Partial cystectomy

## Abstract

**Introduction:**

Inflammatory myofibroblastic tumor (IMT) is a rare form of tumor which is composed of fibroblastic and myofibroblastic spindle cells, with an inflammatory infiltrate of lymphocytes, plasma cells, and eosinophils. IMT may arise from different organs, but it is infrequent in the urinary bladder and usually manifests as haematuria.

**Case presentation:**

Here we report a 20 years old male patient with no previous history of trauma or surgery presented with gross hematuria and severe anaemia. Further workup with transurethral resection biopsy and immunohistochemistry are supportive of IMT and we did partial cystectomy. Follow up cystoscopy after 3 months showed normal bladder wall.

**Clinical discussion:**

An inflammatory myofibroblastic tumor (IMT) is a rare mesenchymal tumor. It comprises differentiated myofibroblastic spindle cells with numerous plasma cells and/or lymphocyte infiltrate. According to the World Health Organisation (WHO) classification, IMT is a low grade or borderline mesenchymal tumor. The commonest site for IMT is the lung, but it rarely occur in the genitourinary tract. Hematuria is the commonest manifestation of bladder IMT. Transurethral resection of bladder tumor (TURBT) and partial cystectomy are treatment modalities.

**Conclusion:**

IMT of the bladder is a rare tumor which manifests mainly with hematuria.

Although TURBT is the standard treatment for IMT, partial cystectomy has lower recurrence rate.

## Highlights

• Inflammatory myofibroblastic tumor (IMT) is a rare form of tumour which is composed of fibroblastic and myofibroblastic spindle cells, with an inflammatory infiltrate of lymphocytes, plasma cells, and eosinophils.
• IMT may arise from different organs and sites, but it is infrequent to arise from the urinary bladder and usually manifests as haematuria.
• The age of the patients diagnosed with bladder IMTs ranged between 3 and 89 years (mean age of 44 years)
• The specific pathogenesis and etiology of IMT remain uncertain and might be connected with the following factors: chronic inflammatory stimulation resulting from bacterial and viral microorganisms (mycobacteria, hepatitis B virus, Corynebacterium, Epstein–Barr virus, EBV, and human papillomavirus), history of bladder trauma or long-term use of hormone therapy, and rearrangements of the anaplastic lymphoma kinase (*ALK*) gene located on chromosome *2p23* (which occur in ∼50% of IMTs).
• Definite diagnostic criteria of IMT are spindle cell proliferation, presence of stellate cells, lymphoplasmacytic infiltrates, and scattered mitoses in myxoid stroma. Immunohistochemical staining may demonstrate positivity for Anaplastic lymphoma kinase (ALK) , Vimentin, SMA, and Cytokeratin. Anaplastic Lymphoma Kinase has been described as a good marker for IMT.
• TURBT is the standard treatment modality but partial cystectomy is a good option with lower recurrence rate.
• IMT has a good prognosis.

## 1 Background

IMTs are lesions of intermediate biologic potential that frequently recur and rarely metastasize. They are composed of myofibroblastic mesenchymal spindle cells accompanied by an inflammatory infiltrate of plasma cells, lymphocytes, and eosinophils. IMTs most frequently occur in the abdominal cavity and lungs, but they may occur in head and neck. This disease has been reported in <1 % of the bladder tumors and is difficult to distinguish because of its non-specific clinical and histological presentation [1]. In this case report we present a 20 years old male patient presented with gross haematuria and diagnosed to have IMT and treated with partial cystectomy.

## 2 Case presentation

20 years old male patient from Somali region of Ethiopia referred to our hospital with gross hematuria of two months duration. Associated to this he had easy fatigability and tinnitus. No history of trauma or surgical procedure. He is not smoker. No family history of similar illness.

On physical examination he had tachycardia to the level of 120 bits per minute, blood pressure was in the normal range and he was afebrile. He had paper white conjunctiva and non-icteric sclera. On chest examination there was clear and good air entry bilaterally. No palpable abdominal mass.

Even though it is rare in this age group, our provisional diagnosis was bleeding bladder tumor and we investigated him with complete blood count which showed hemoglobin of 6.1 g/dl but other parameters were in the normal range.

**Fig. 1:**
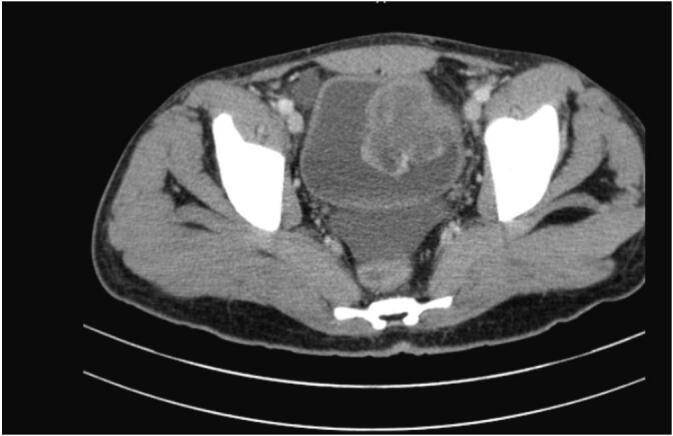
CECT (left antero-laterral bladder wall heterogenously enhancing mass).

Renal function test and liver function test were in normal range. Urine dipstick showed blood plus four (+4). He had also abdominopelvic contrast enhanced computed tomography (CECT) scan from the referral which showed 4.8 cm∗5 cm heterogeneously enhancing mass on the left anterolateral bladder wall (Fig. 1). Cystoscopy showed polypoid solid bladder mass on the left anterolateral bladder wall with active bleeding. With a diagnosis of severe anaemia secondary to bleeding bladder tumor he was resuscitated, transfused with 5 units of red blood cell. Subsequently transurethral resection biopsy was taken and bleeding was controlled with coagulation. Histopathology result was myxoid mesenchyemal tumor and immunohistochemistry (IHC) was supportive of inflammatory myofibroblastic tumor of the bladder (desmin, vimentin and CD34 were positive) whereas myogenin and CK AE1/AE3 were negative.

**Fig. 2:**
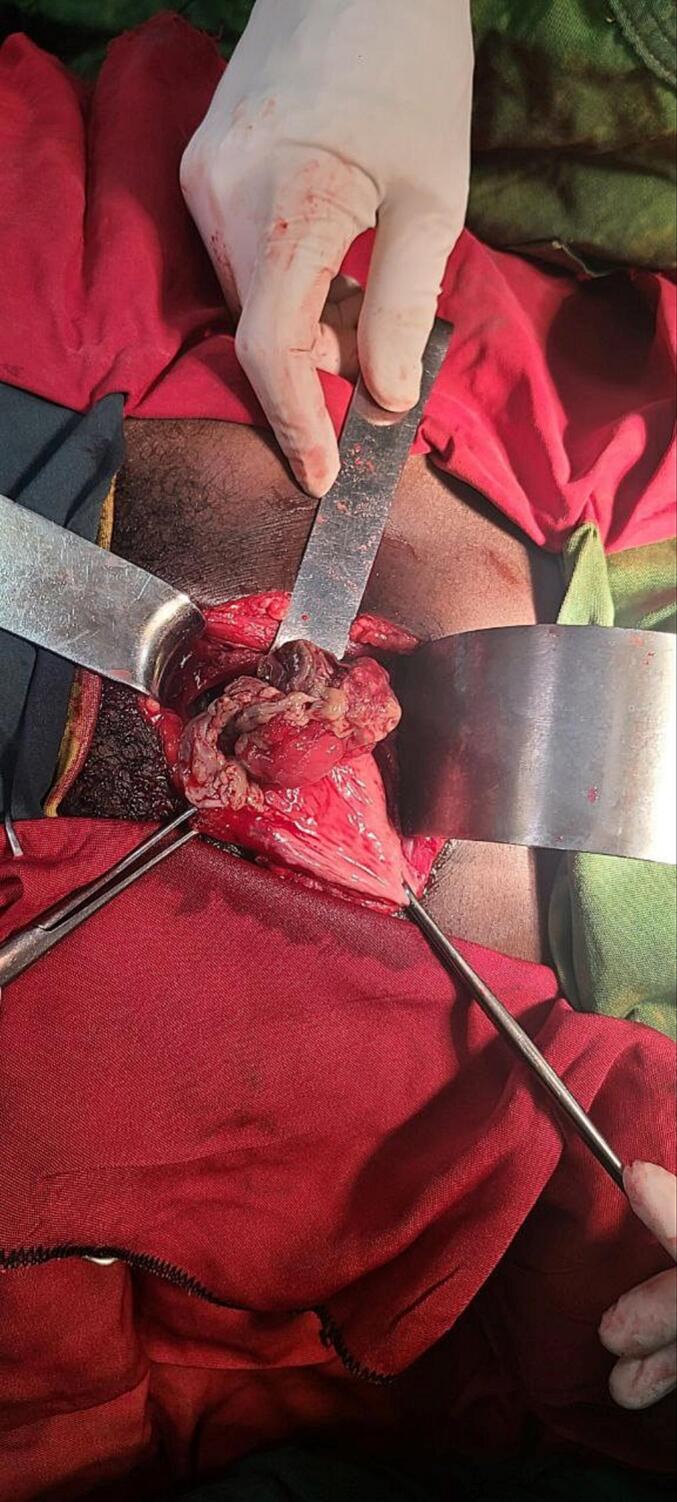
Intra operative image of the tumor (fungated with overlying necrotic tissue).

**Fig. 3:**
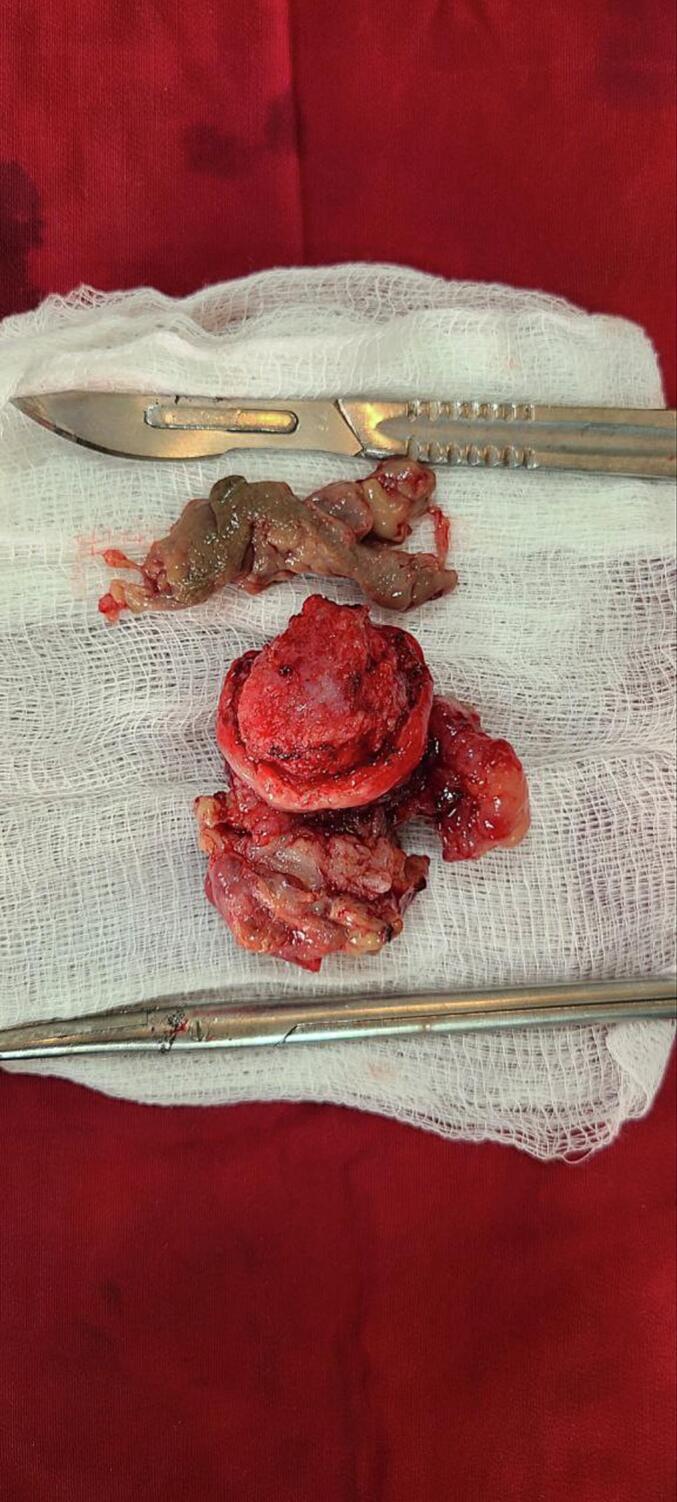
Excised tumor and necrotic tissue.

**Fig. 4:**
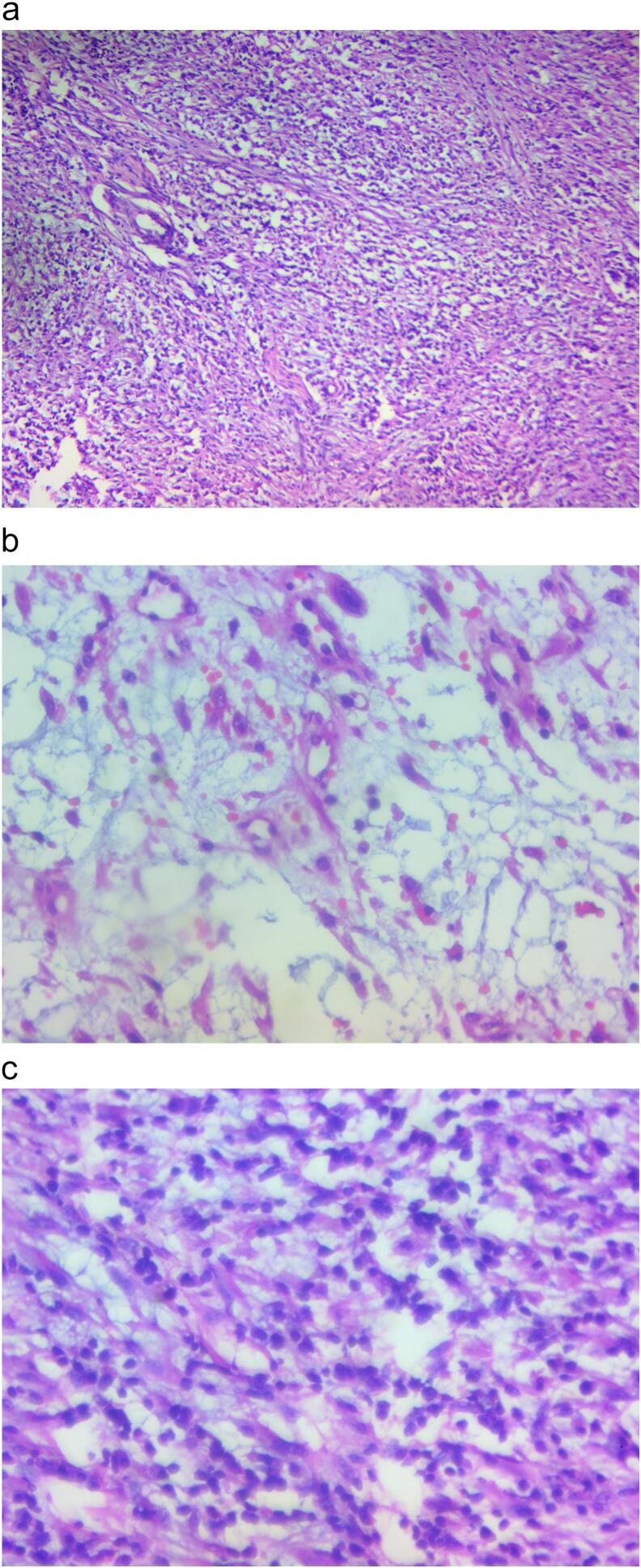
Histology of the tumor.

Subsequently partial cystectomy was decided; with vertical lower mid line incision peritoneal cavity entered and bladder was accessed. Bladder was opened vertically at midline. There was 5.5 cm by 5 cm fungated, solid mass at the left anterolateral bladder wall with necrotic overlying surface (Fig. 2). There was no active bleeding. No tumor on other site of the bladder and ureteric orifices were visible bilaterally. No lymphadenopathy. Tumor resected circumferentially two centimetres away from the tumor (Fig. 3). Trans urethral catheter inserted and bladder closed in layers. Drain also placed at the retro pubic area. Subsequently drain output was minimal on the first post operative day and it was removed. He was discharged on the second post-operative day. Transurethral catheter was removed after 7 days. The excisional biopsy result showed spindle cell neoplasm in favour of inflammatory myofibrobla tumor (Fig. 4a,b,c), tumor margins were free. Surveillance cystoscopy was done 3 months post operatively and there was no growth in the bladder.

The patient had no any lower urinary tract symptoms, bladder capacity was measured 4 months after the surgery and it was normal (300 ml).

## 3 Discussion

An inflammatory myofibroblastic tumor (IMT) is a rare mesenchymal tumor, first reported in 1939. It was misdiagnosed as a highly malignant sarcoma [2]. It comprises differentiated myofibroblastic spindle cells with numerous plasma cells and/or lymphocyte infiltrates [2,3]. According to the World Health Organisation (WHO) classification, IMT is a low grade or borderline mesenchymal tumor [2].

The commonest site for IMT is the lung. It also presents commonly in visceral organs and deep soft tissues of the abdomen, pelvis, retroperitoneum, head and neck. Any part of the human body may be affected, including somatic soft tissues, bone, extremities, larynx, or even central nervous system. It can rarely manifest in the esophagus, the pericardium, the heart, the spinal meninges, and the adrenal glands [2]. An IMT can occur in the genitourinary system, but it is most common in the bladder and accounts for <1 % of all bladder tumours [5]. The first report of IMT of the urinary bladder was made in 1980 [3]. The origin of IMT is controversial, but a recent report suggests that it is neoplastic because of aggressive behaviour, involvement of chromosome 2p23, and congenital clonality [4].

IMT also known as pseudo sarcoma, atypical fibromyxoid tumor, atypical myofibroblastic and plasma cell granuloma [4].

IMTs occur at a relatively younger age than the urothelial carcinoma. Age of the patients diagnosed with bladder IMTs ranged between 3 and 89 years (mean age of 44 years), and gross hematuria is the commonest presentation in >60 % of the cases [1]. It is diagnosed with a slight predominance of females to men [2,3].

The specific pathogenesis and etiology of IMT is not well known. But it might be connected with the following factors: chronic inflammatory stimulation resulting from bacterial and viral microorganisms (mycobacteria, hepatitis B virus, Corynebacterium, Epstein–Barr virus, EBV, and human papillomavirus), history of bladder trauma or long-term use of hormone therapy, and rearrangements of the anaplastic lymphoma kinase (*ALK*) gene located on chromosome *2p23* (which occur in ∼50 % of IMTs) [5].

The most frequently reported symptom is gross haematuria followed by dysuria and increased urinary frequency. Contrast enhanced CT shows polypoid nodules on the bladder walls with ring enhancement [6]. Well-defined tumours are considered to be a feature of the bladder IMT on CT imaging [7]. Our patient was presented with gross hematuria with severe anaemia which requires transfusion of five units of packed red blood cell. Both epithelial and myogenic markers can be expressed in IMT and may lead to a misdiagnosis of sarcomatoid carcinoma, leiomyosarcoma, and rhabdomyosarcoma [4].

Definite diagnostic criteria of IMT are spindle cell proliferation, presence of stellate cells, lymphoplasmacytic infiltrates, and scattered mitoses in myxoid stroma [4]. Immunohistochemical staining may demonstrate positivity for Anaplastic Lymphoma Kinase (ALK), Vimentin, SMA, and Cytokeratin. Anaplastic Lymphoma Kinase has been described as a good marker for IMT. Myogenin, a potent marker for rhabdomyosarcoma, helped in exclusion of this tumors [4]. In our case, it was positive for vimentin(diffuse), desmine(patchy) and CD34; whereas myogenin and CK AE1/AE3 were negative.

The standard treatment for IMT is TURBT [8,9]. Although TURBT has the advantages of less trauma and faster recovery, a multicentre study showed that the rate of second surgery after surgery was 21 %, markedly higher than the rate of 2 % in patients who underwent partial cystectomy [7]. In our case first we did TURBT to stop bleeding and to take biopsy. Subsequently we did partial cystectomy. Surveillance cystoscopy after 3 months shows normal bladder mucosa. Partial cystectomy for large tumor may compromise the bladder capacity. In such cases augmentation cystoplasty may be needed. In our case the tumor size relative to the bladder size is not that much large. And resection with adequate tumor margin did not affect the bladder capacity.

The long-term prognosis of bladder IMT is good. The recurrence rate ranges between 9 % and 21 %, and the metastasis rate is about 4 % [1]. IMT has a relatively good prognosis and is considered to be a tumor with intermediate biologic potential because of its low risk of distant metastases [3].

This case report is written based on SCARE guideline [10].

## 4 Conclusion

IMT of the bladder is a rare form of tumor. It comprises differentiated myofibroblastic spindle cells with numerous plasma cells and/or lymphocyte infiltrates. According to the World Health Organisation (WHO) classification, IMT is a low grade or borderline mesenchymal tumor. Haematuria is the commonest presentation. Cystoscopy, CT scan, transurethral resection biopsy and immunohistochemistry are the diagnostic modalities. TURBT and partial cystectomy are the treatment modalities. IMT has a good prognosis.

## Abbreviations

IMT: Inflammatory myofibroblastic tumor
ALK: anaplastic lymphoma kinase
CECT: contrast enhanced computed tomography
CK AE1/AE3: cytokeratin AE1/AE3
TURBT: transurethral resection of bladder tumor

## Clinical trial number

N/A.

## CRediT authorship contribution statement

1: Chale Yohannes Tegegne (urology resident): Operated on the patient, conceived, wrote the original draft, edited and submitted the report.

2: Mekuanint Asfaw Yitayew (urologist): Operated on the patient, edited the report

3: Chalachew Tenna Alemu( urology resident): Operated on the patient and participated on writing the draft of the report.

4: Samuel Fekadu Shiferaw(urology resident): Did the TURBT, participated on editing the report and patient followup

5: Admassu Melaku Mikru(urologist): Edited the report

6: Abdiqadir Omer Rabile(pathology resident): Evaluated the specimen, contributed the histologic images.

## Patient consent

Written informed consent was obtained from the patient for publication of this case report and accompanying images. A copy of the written consent is available for review by the Editor-in-Chief of this journal on request.

## Ethical approval

Ethical approval was provided by the authors' institution.

## Guarantor

Chale Yohannes Tegegne (urology resident).

## Funding

N/A.

## Registration of Research Studies

N/A.

## Declaration of competing interest

N/A.

## Data Availability

The data set used and/or analysed during this case report are available from the corresponding author on request.

## References

[1] Siemion K., Reszec-Gielazyn J., Kisluk J., Roszkowiak L., Zak J., Korzynska A. (2022). What do we know about inflammatory myofibroblastic tumors? - a systematic review. Adv. Med. Sci..

[2] Sbaraglia M., Bellan E., Dei Tos A.P. (2021). The 2020 WHO classification of soft tissue Tumours: news and perspectives. Pathologica.

[3] Daoqing Song a Wja, b, Ze Gao a, Ningning Liu c, Shimin Zhang a, Yuqian Zong d, Zhiqing Fang a,∗, Yidong Fan. <Inflammatory myofibroblastic tumor of urinary bladder with severe hematuria_ A Case report and literature review - PMC.pdf>. 10.1097/MD.0000000000013987 PMC6344176 30608442

[4] Jones E.C., Clement P.B., Young R.H. (1993). Inflammatory pseudotumor of the urinary bladder. A clinicopathological, immunohistochemical, ultrastructural, and flow cytometric study of 13 cases. Am. J. Surg. Pathol..

[5] Suer E., Gulpinar O., Mermerkaya M., Burgu B., Celepli P., Sertcelik A. (2012). Inflammatory myofibroblastic tumor of the bladder in a 10-year-old girl. Urology.

[6] Yoshitaka Furukawa a KKa, *, Hisashi Komoto a, Masahiro Zenitani b, Takaharu Oue b, Hiroyuki, Yokoyama a YIa, Takashi Yamasaki c, Seiichi Hirota c, Koichiro Yamakado a. <CT and MRI Findings of Inflammatory Myofibroblastic Tumor in the Bladder - PMC.pdf>. 10.1159/000521921 PMC8921957 35350811

[7] Chen B., Li S., Fang X., Xu H., Yu J., Liu L. (2020). Inflammatory Myofibroblastic tumor of the urinary system on computed tomography at a high-volume institution in China. Urol. Int..

[8] Roth J.A. (1980). Reactive pseudosarcomatous response in urinary bladder. Urology.

[9] Elizabeth A. Montgomery M, Dawn D. Shuster, BS, Ashlie L. Burkart, MD, Jose M. Esteban M, Anita Sgrignoli, MD, Lori Elwood, MD, David J. Vaughn, MD, Constance A. Griffin M, and Jonathan I. Epstein, MD. Inflammatory Myofibroblastic Tumors of the Urinary Tract: A Clinicopathologic Study of 46 Cases, Including a Malignant Example Inflammatory Fibrosarcoma and a Subset Associated With High-grade Urothelial Carcinoma. 10.1097/01.pas.0000213280.35413.1b 17122505

[10] Sohrabi C., Mathew G., Maria N., Kerwan A., Franchi T., Agha R.A., Collaborators. (2023 May 1). The SCARE 2023 guideline: updating consensus Surgical CAse REport (SCARE) guidelines. Int. J. Surg..

